# Distribution and Antimicrobial Resistance Patterns of Critical and High-Priority Gram-Negative Pathogens Isolated from Endotracheal Aspirates in an Intensive Care Unit

**DOI:** 10.3390/microorganisms14102281

**Published:** 2026-10-07

**Authors:** Andreea-Loredana Golli, Flavia Liliana Turcu, Ovidiu Mircea Zlatian, Simona Georgiana Popa, Maria Stoica

**Affiliations:** 1 Department of Public Health and Management, University of Medicine and Pharmacy of Craiova, 200349 Craiova, Romania; andreea.golli@umfcv.ro; 2 Department of Nephrology & Dialysis, Carol Davila University of Medicine and Pharmacy Bucharest, 050474 Bucharest, Romania; 3 Department of Microbiology, University of Medicine and Pharmacy of Craiova, 200349 Craiova, Romania; 4 Department of Diabetes, Nutrition and Metabolic Diseases, University of Medicine and Pharmacy of Craiova, 200349 Craiova, Romania; 5 Department of Intensive Care, University of Medicine and Pharmacy of Craiova, 200349 Craiova, Romania

**Keywords:** critical pathogens, high priority pathogens, Gram-negative pathogens, multidrug-resistance

## Abstract

Lower respiratory tract infections represent the world’s deadliest infectious disease, responsible for approximately 2.5 million deaths worldwide. The aim of the study was to determine the distribution and antimicrobial susceptibility profiles of the main critical and high priority Gram-negative bacteria isolated from endotracheal aspirates obtained from Intensive Care Unit (ICU) patients. We conducted a four-year microbiological surveillance study (2022–2025) in a tertiary Romanian ICU. A total of 1529 Gram-negative bacterial isolates were detected in 1391 endotracheal aspirates. Most frequently reported were *Pseudomonas aeruginosa* (422–27.60%), *Acinetobacter baumannii* (296–19.36%), *Escherichia coli* (274–17.92%) and *Klebsiella pneumoniae* (271–17.73%). The prevalence of MDR *Acinetobacter baumannii* declined to approximately 40% in 2025, whereas XDR isolates increased to 13.51%, twice the 2022 value. Carbapenem resistance among *Acinetobacter baumannii* remained high (around 70%) in 2025, despite a significant decline from 2022 (*p* < 0.001). Approximately 65% of *K. pneumoniae* isolates were MDR/XDR, with an increase in carbapenem resistance. MDR *Pseudomonas aeruginosa* increased significantly from 14.14% in 2022 to 39.84% in 2025 (*p* < 0.001) and a significant upward trend in carbapenem resistance was registered, exceeding 70% for imipenem in 2025. These findings underscore the need for continuous microbiological and epidemiological surveillance and targeted interventions to prevent the spread of highly resistant pathogens.

## 1. Introduction

According to the analysis from the Global Burden of Diseases, Injuries, and Risk Factors Study (GBD) 2023, lower respiratory tract infections (LRTIs) remain the world’s leading infectious cause of death [1], being responsible for an estimated 2.50 million deaths worldwide each year and nearly 99 million disability-adjusted life-years (DALYs), with the majority of deaths recorded at extreme ages (children younger than 5 years and adults over 70 years). From the point of view of etiology, respiratory infections caused by multidrug-resistant (MDR) Gram-negative pathogens represent a major clinical challenge, especially among hospitalized and critically ill patients [2], accounting for 1.5 million deaths and over 75,000,000 disability-adjusted life-years in 2019. These pathogens are more likely to be resistant to antibiotics in Southern Europe, compared to other regions of the continent [3].

Among LRTIs, healthcare-associated pneumonia, including hospital-acquired pneumonia (HAP) and ventilator-associated pneumonia (VAP), is among the most common and serious nosocomial infections, particularly in critically ill and mechanically ventilated patients. These infections are of particular importance due to the high risk of mortality, prolonged stay in the intensive care unit (ICU), increasing antibiotic consumption, and higher costs of medical care [4]. *Acinetobacter baumannii*, *Pseudomonas aeruginosa*, *Klebsiella pneumoniae,* and *Escherichia coli* are the most frequently involved Gram-negative pathogens in the etiology of these infections in critically ill patients admitted to the ICU and, especially, in those mechanically ventilated [5].

The diagnosis of LRTIs is based on the integration of clinical, radiological, and microbiological findings. In mechanically ventilated patients, endotracheal aspirate represents the most frequently collected respiratory specimen for microbiological culture and is routinely used to identify potential respiratory pathogens [6]. According to the guideline developed in 2017 by the European Respiratory Society/European Society of Intensive Care Medicine/European Society of Clinical Microbiology and Infectious Diseases/Asociación Latinoamericana del Tórax (ERS/ESICM/ESCMID/ALT) [7], in critically ill VAP patients, the clinical benefits of invasive diagnostic techniques remain less well established, given the potential adverse effects of bronchoscopy on gas exchange. For patients with suspected hospital-acquired (HAP) and ventilator-associated pneumonia (VAP), the method recommended for the diagnosis by the Infectious Diseases Society of America and the American Thoracic Society is noninvasive sampling (endotracheal aspiration) with semiquantitative cultures [8].

MDR pathogens, especially Gram-negative bacteria, are the most frequent microorganisms involved in the lower respiratory infections in ICU patients requiring invasive mechanical ventilation [5].

Increasing resistance of the Gram-negative pathogens, including *Acinetobacter* spp., *Escherichia coli (E. coli*) and *Klebsiella pneumoniae* to carbapenems and fluoroquinolones, essential for the treatment of severe infections, is a problem of great concern [9]. According to WHO Report 2025 [9], the fatality rate associated with infections with carbapenem-resistant *Klebsiella pneumoniae* and *Acinetobacter* spp. exceeds 30%.

The continued emergence and spread of resistant microorganisms lead to the decreasing availability of therapeutic resources that can be used for the treatment of the infectious diseases caused by MDR pathogens [10].

The global burden of antimicrobial resistance (AMR) continues to increase, making it a major contributor to mortality worldwide; recent analyses estimate that bacterial AMR contributed to approximately 1.14 million deaths in 2021 [9] and is projected to claim 1.91 million global lives in 2050 [11], with Romania being one of 15 countries that reported AMR percentages equal to or above 50% [12].

According to the 2024 World Health Organization (WHO) Bacterial Priority Pathogens List (BPPL) [13], Gram-negative pathogens that are resistant to last-resort antibiotics, such as *Acinetobacter baumannii* carbapenem-resistant and Enterobacterales third-generation cephalosporin-resistant and carbapenem-resistant are classified as critical priority pathogens. Also, *Pseudomonas aeruginosa* carbapenem-resistant is included in the high-priority pathogens [13].

Difficult-to-treat Gram-negative pathogens increasingly drive the burden of antimicrobial resistance in the WHO European Region. Recent analyses from the WHO Global Antimicrobial Resistance and Use Surveillance System (GLASS) [9] indicate that *E coli* and *Klebsiella pneumoniae* continue to be the predominant contributors to the global resistance burden, exhibiting high rates of resistance to third-generation cephalosporins and a persistent increase in carbapenem-resistant *Klebsiella pneumoniae*. Furthermore, carbapenem-resistant *Acinetobacter baumannii* remains a major concern in several countries within the Region, where invasive infections caused by this pathogen are associated with substantial mortality.

These data underline the need for early detection and surveillance of multidrug-resistant pathogens, given the progressive reduction in effective therapeutic options [14,15]. An extremely vulnerable category of patients is represented by critically ill patients admitted to intensive care units, who are exposed to an increased risk of developing severe lower respiratory tract infections due to extended exposure to invasive medical devices, multiple underlying comorbidities, prolonged hospitalization and long-term antibiotic administration.

There is little data available from Eastern European ICUs about hospital-acquired and ventilator-associated pneumonia. Romania is one of the EU countries that report a high prevalence of MDR pathogens [4], with alarming multidrug and carbapenem resistance rates among clinical *Acinetobacter baumannii* isolates, particularly within intensive care units [15].

Knowledge of the local microbiological profile and antimicrobial resistance patterns of critical and high-priority Gram-negative pathogens isolated from endotracheal aspirates can help improve the antibiotic stewardship policies, and implement effective infection control practices.

The present study aimed primarily to evaluate the distribution and antimicrobial resistance patterns of critical and high-priority Gram-negative bacteria isolated from endotracheal aspirates over a 4-year period (2022–2025) in patients admitted to the ICU of a tertiary-care hospital from South-West Romania. The second objective was to perform a comparative analysis to assess the trend of AMR during the study period, which can contribute to the improvement of strategies for the prevention and control of infections with MDR Gram-negative pathogens.

## 2. Materials and Methods

This research is an extensive four-year retrospective analysis of endotracheal aspirate samples collected from critically ill patients over 18 years old who were admitted to the ICU of the Emergency Clinical County Hospital of Craiova, Romania. The hospital provides intensive care for highly complex medical and surgical cases from Dolj County and the South-West Region of Romania, requiring invasive procedures and prolonged antimicrobial treatment.

The analysis included existing data from the Clinical Microbiology Laboratory database regarding positive bacterial cultures from the respiratory tract in which Gram-negative pathogens have been identified in patients admitted between January 2022 and December 2025. Bacterial duplicates, defined as the same microorganism with an identical antimicrobial resistance profile isolated from the same patient, were excluded. Only the first isolate with the same pathogen was included. If during the study period, there were several endotracheal aspirates collected from the same patient, which highlighted different pathogens, they were included in the analysis.

The samples were collected in sterile containers from mechanically ventilated patients with clinical suspicion of lower respiratory tract infection and immediately transported to the microbiology laboratory, according to the hospital procedures. Clinically, the patient was required to have at least one systemic feature, including fever greater than 38 °C, without other causes, leukopenia (<4000/mm^3^) or leukocytosis (≥12,000/mm^3^). Respiratory features included new onset or change in respiratory secretion characteristics (color, smell, quantity, and consistency), worsening gas exchange (hypoxemia or increase in the concentration of the inspired mixture in oxygen and/or increase in respiratory volume/minute).

The specimens were cultured on blood agar Columbia and MacConkey agar plates and incubated aerobically at 37 °C for 24 h. Endotracheal aspirates showing less than 10^4^ CFU/mL by semiquantitative culture were considered commensal or contaminant and were excluded, according to the laboratory procedure. Quantitative culture was performed using a calibrated 1 µL (0.001 mL) inoculation loop, a standard technique for semi-quantitative bacterial enumeration. Because each colony-forming unit recovered from a 1 µL inoculum corresponds to 1000 CFU/mL of original specimen, colony counts were multiplied by this dilution factor (1000) to calculate CFU/mL. Samples yielding ≥10 colonies on the primary isolation plate were therefore classified as ≥10^4^ CFU/mL. Automated identification was conducted using the Vitek 2 Compact system (Biomerieux, Durham, NC, USA). The following antimicrobial-susceptibility cards were used: AST-XN26, AST-N438 and AST-N427. For the analysis of the resistance profile of Gram-negative pathogens framed in the critical and high-priority group, we included the following antibiotics in the present study: ceftriaxone (CRO), cefotaxime (CTX), ceftazidime (CAZ), cefepime (FEP), ciprofloxacin (CIP), levofloxacin (LEV), gentamicin (GEN), piperacillin–tazobactam (TPZ), imipenem (IPM), meropenem (MEM), tigecycline (TGC), and colistin.

Quality control for antimicrobial susceptibility testing was performed using standard American Type Culture Collection (ATCC) strains such as *Stenotrophomonas maltophilia* ATCC 17666 and *Pseudomonas aeruginosa* ATCC 27853 for non-fermentative Gram-negative bacilli and *Escherichia coli* ATCC 25922 and *Enterobacter hormaechei* ATCC 700323 for other Gram-negative pathogens.

Broth microdilution was used for colistin testing, using the ComASP Colistin Test Panel (Liofilchem, Roseto degli Abruzzi, Italy). The antimicrobial susceptibility test was carried out according to Clinical Laboratory Standard Institute (CLSI) guidelines corresponding to the year involved [16,17,18,19]. Colistin interpretative breakpoints used for validation of resistance were >2 μg/mL for Enterobacterales and *Acinetobacter baumanni* and >4 μg/mL for *Pseudomonas aeruginosa.*

Information on patients’ age, sex, hospital department, specimen source, and antimicrobial susceptibility patterns of Gram-negative isolates was retrieved from the hospital information system and analyzed using Microsoft Excel (2024). No special informed consent from patients/legal representatives was required, as it is a retrospective study, and each patient/legal representative signs at admission a consent form that specifies the agreement to carry out investigations and treatment, as well as to participate in medical research, according to the procedure of our hospital.

The prevalence of Gram-negative microorganisms was summarized as percentages.

The resistance rates were calculated as the proportion of resistant Gram-negative isolates among all tested Gram-negative isolates. Antibiotics for which the number of tested strains was insufficient according to the CLSI guidelines (minimum of 30 isolates per organism and/or organism group) [16,20] were excluded from the analysis. In the case of Gram-negative pathogens for which fewer than 30 strains were identified, the percentage of those tested exceeded 90% for at least one of the representatives of the class of antibiotics for which the interpretation was made. Intermediate results are signaling that high-dose combination strategies are mandatory if used and were considered susceptible at a higher dose exposure, with the recommendation of adjustment of the therapeutic dose by the treating physician.

Because the study addresses Gram-negative microorganisms classified according to the WHO BPPL list 2024 [13] in the critical priority group (*Acinetobacter baumannii* carbapenem-resistant, Enterobacterales third-generation cephalosporin-resistant, and Enterobacterales carbapenem-resistant) and high-priority group (*Pseudomonas aeruginosa* carbapenem-resistant), a pertinent analysis of the obtained results was carried out.

For the evaluation of the antibiotic-resistance trend over the study period, comparative analysis was carried out for the main isolated critical and high-priority Gram-negative pathogens, individually for each combination of antibiotics and pathogens, using the chi-square test for independence or Fisher’s exact test for small groups. The chi-framework test was used to compare the differences among antibiotic resistance rates over the years considered in the study, and chi-square for linear trend (extended Mantel–Haenszel) was used for analysis of linear trends in proportions, only for antibiotics that showed statistically significant differences in the distribution of resistance during the years under consideration. The resistance rates for each of the main pathogens analyzed were compared for each year compared to the previous year and between the first and last years of the study period. The Holm correction for multiple comparisons was applied.

Statistical analyses were performed using Epi Info version 7.2.4.0. A two-sided *p*-value < 0.05 was considered to be statistically significant.

The percentages of MDR, extensively drug-resistant (XDR), and pan-drug-resistant (PDR) Gram-negative pathogens were analyzed. For the assessment of multidrug resistance, resistance to at least three distinct classes of antibiotics—aminoglycosides, cephalosporins, carbapenems, tetracyclines, or fluoroquinolones—has been taken into account [21]. XDR isolates were defined as those exhibiting resistance to at least one antimicrobial agent in all but two or fewer antimicrobial categories, thereby retaining susceptibility to only one or two antimicrobial classes. PDR isolates were defined as those exhibiting resistance to all antimicrobial agents across all antimicrobial categories tested in the hospital, in accordance with the methodology for the surveillance and control of healthcare-associated infections, developed by the Ministry of Health of Romania [22].

During the study period, there were no changes in the procedures that specified diagnostic criteria, laboratory specimen collection methods, or culture methodologies. At the end of 2023, however, a procedure was developed for screening patients to detect those colonized/infected with MDR pathogens, along with the isolation guide for infected patients based on the route of transmission; both are mandatory procedures aimed at preventing the transmission of healthcare-associated infections, in accordance with existing legislation in Romania [23]. The authors followed the STROBE Guidelines during the conduct of this study [24].

## 3. Results

### 3.1. Distribution of the Main Isolates

During a four-year period (2022 to 2025), a total of 5751 samples of endotracheal aspirates were collected from mechanically ventilated patients through an endotracheal or tracheostomy tube. Among these, 48.28% (2777) were positive (Table 1) while in 2974 samples (51.71%) no pathogen was identified.

**Table 1:** Distribution of endotracheal aspirates according to the isolated pathogen.

Year	Microorganism		Total
	Gram Negative	Gram Positive	
2022	322	547	869
2023	377	386	763
2024	349	190	539
2025	343	263	606
Total	1391	1386	2777

Gram-negative pathogens were isolated in 1391 samples collected from 1334 patients, of which, 57 patients contributed more than one specimen. Monomicrobial growth was found in 90.36% of the samples (1257), whereas 9.64% were polymicrobial.

The majority of the samples in which Gram-negative pathogens were found were obtained from patients >65 years (984/1391—70.74%) and male patients (861). Most of the samples were collected in 2023 (377/1391—27.10%) (Table 2).

**Figure 1:**
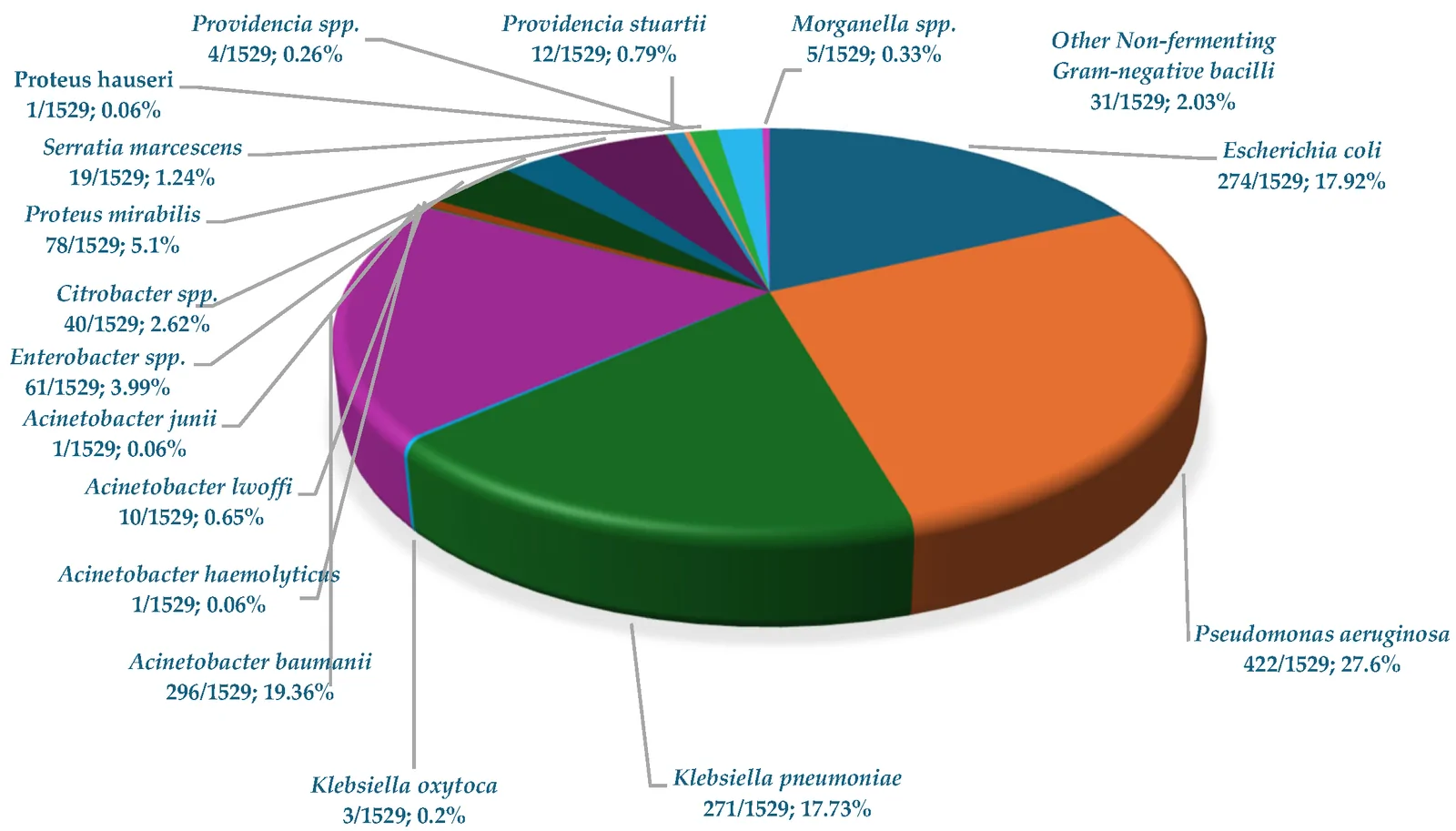
Distribution of Gram-negative pathogens isolated from endotracheal aspirates collected from ICU patients, from 2022 to 2025.

Given that Gram-negative pathogens are the most frequent MDR microorganisms identified in respiratory tract samples from ICU patients, the present analysis focused on the distribution and antimicrobial resistance patterns of critical and high-priority Gram-negative microorganisms isolated from respiratory tract specimens. A total of 1529 Gram-negative bacterial pathogens have been identified between 2022 and 2025, excluding cases where there was more than one isolate of the same pathogen from the same patient in the same year Almost 28% of the pathogens (425/1529) were isolated in the samples collected in 2023 and around 24% in the other three years. *Pseudomonas aeruginosa* was the most prevalent pathogen (422–27.60%), followed by *Acinetobacter baumannii* (296–19.36%), *E. coli* (274–17.92%), and *Klebsiella pneumoniae* (271–17.73%) (Figure 1).

However, the analysis of the distribution of pathogens for each year revealed a different hierarchy. Thus, *Pseudomonas aeruginosa* ranked first among the isolated pathogens in 2024 (97/374—25.94%) and 2025 (123/368—33.42%), and 2nd in 2022 (99/362—27.35%) and 2023 (103/425—24.24%). The increase recorded in the second part of the studied interval was statistically significant (*p* = 0.03) (Table S1). *Acinetobacter baumannii* ranked first in frequency in 2022 (105/362—29%), but the number of isolated strains decreased to approximately one-third in 2025 (37/368—10.06%, *p* < 0.001), occupying 3rd place in the hierarchy. The number of *Klebsiella pneumoniae* strains was almost the same in 2022 (50-13.81%), 2024 (58-15.51%) and 2025 (51-13.86%), ranking 4th and 3rd respectively. However, special attention is drawn to the significant increase in the number of isolated strains in 2023 (112-26.35%, *p* < 0.001), placing this pathogen at the top of the hierarchy in that year. An ascendant trend was highlighted between 2022 and 2025 in the percentage of *E. coli* (*p* = 0.05), ranking 3rd place in 2022 (55/362—15.19%), 4th in 2023 (68/425—16%), and 2nd in 2024 (74/374—19.79%) and 2025 (77/368—20.92%) (Table S1).

Less frequently isolated Gram-negative pathogens include *Proteus mirabilis* (5.10%), *Enterobacter* spp. (3.99%), *Citrobacter* spp. (2.62%), and other non-fermenting Gram-negative bacilli (2.03%) (Figure 1).

### 3.2. Antimicrobial Resistance in Main Bacterial Gram-Negative Species

#### 3.2.1. *Acinetobacter baumannii*

**Figure 2:**
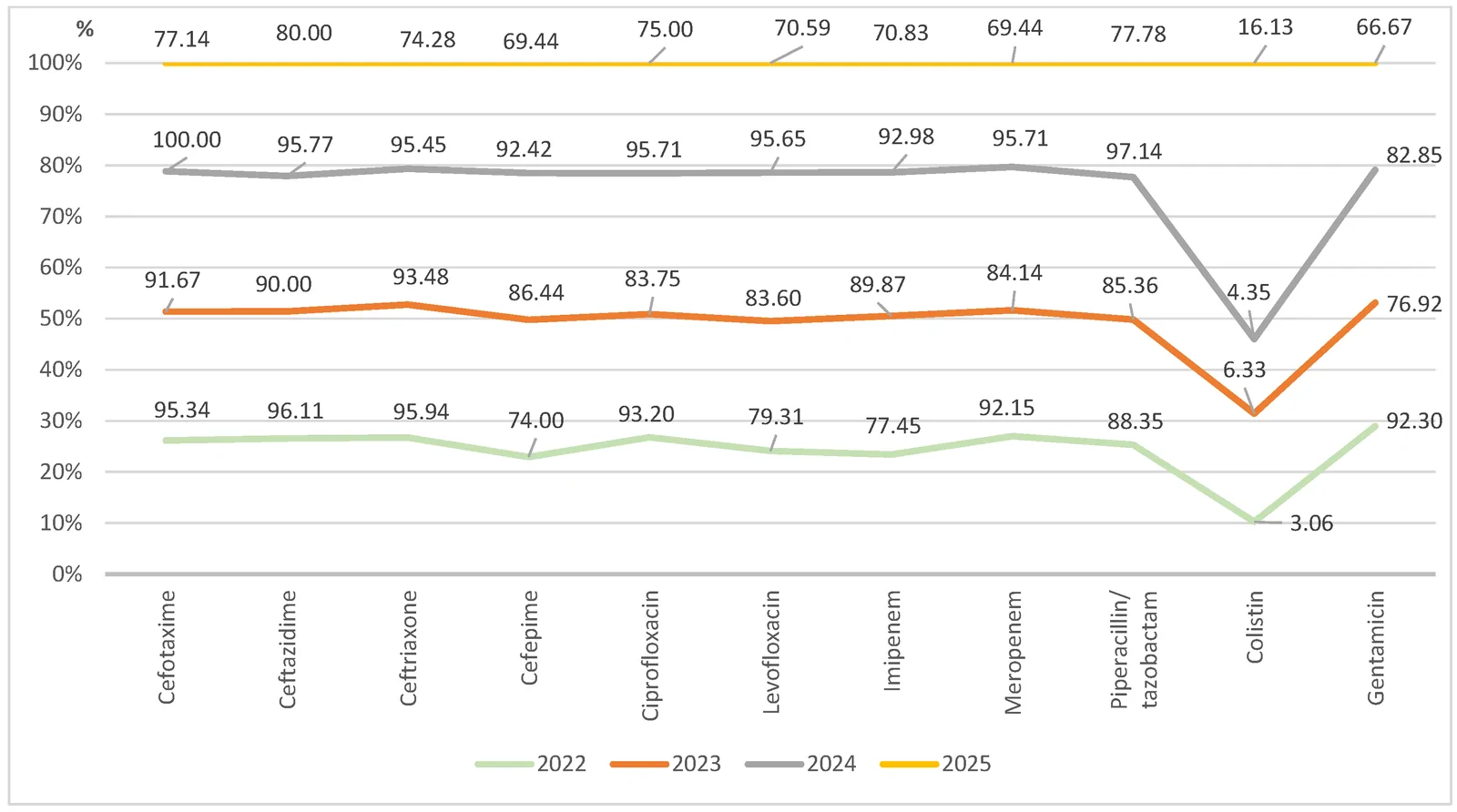
Antimicrobial resistance profile of *Acinetobacter baumannii* isolated from endotracheal aspirates, 2022–2025.

*Acinetobacter baumannii* was the second most frequently isolated, representing around 20% of all the Gram-negative strains (296/1529). Overall, during 2022–2025, the antimicrobial resistance profile of *Acinetobacter baumannii* revealed a very high level of resistance for the third-generation cephalosporins (cefotaxime: 236/254—92.91%; ceftazidime: 267/289—92.38%; ceftriaxone: 182/199—91.46%) and carbapenems (imipenem: 220/262—83.97% and meropenem: 255/290—87.93%) (Table S2). Also, over 80% of the strains were resistant to fourth-generation cephalosporins (cefepime -211/261), aminoglycosides (gentamycin: 238/288—82.64%), fluoroquinolones (ciprofloxacin: 254/285—89.12%, levofloxacin: 210/251—83.66%), and piperacillin–tazobactam (257/291—88.31%) (Table S2). Almost 6% of the tested strains were resistant to colistin (Table S2). However, during the analyzed period, both the number of isolated strains of *Acinetobacter baumannii* and their resistance to most of the antibiotics for which they were tested decreased significantly. Although in the first three years of the study, the resistance to third-generation cephalosporins remained very high, exceeding 90%, the year 2025 marked a significant decrease compared to 2022 for cefotaxime (*p* = 0.006; OR = 6.07; 95% CI: 1.69–21.77), ceftazidime (*p* = 0.007; OR = 2.55; 95% CI:1.68–22.66), and ceftriaxone (*p* = 0.002; OR = 8.19; 95% CI:2.05–32.62) (Figure 2, Table S3). The comparative analysis of *Acinetobacter baumannii* resistance to cefotaxime showed a statistically significant decrease in the proportion of resistant strains between 2024 and 2025 (*p* = 0.0004) as well as between 2022 and 2025 (*p* = 0.006). After applying the Holm correction for multiple comparisons, the adjusted *p*-values were 0.0016 and 0.018, respectively, both differences maintaining their statistical significance. The proportion of resistant *Acinetobacter baumanni* strains to ceftazidime showed a significant decrease (*p* = 0.007) between 2022 (96.11%) to 2025 (80%), the difference remaining statistically significant after adjusting for multiple comparisons (*p* = 0.028). The percentage of resistant strains was also significantly lower for ceftriaxone in 2025 (74.28%) compared to 2022 (95.24%, *p* = 0.002), and 2024 (95.45%, *p* = 0.01), differences that remained significant even after the application of the Holm correction (*p* = 0.008, respective *p* = 0.030).

In the case of carbapenem resistance, the study highlighted a significant increase for imipenem in 2023, reaching almost 90% (71/79) compared to 2022 (79/102; 77.45%, *p* = 0.04), an increase that continued in 2024 (53/57; 92.98%). A significant decrease was recorded in 2025 (17/24; 70.83%, *p* = 0.02), but the number of the isolated *Acinetobacter baumannii* strains as well as those tested was much lower. After applying the Holm correction, none of the analyzed differences remained statistically significant. In the case of meropenem, the percentage of resistant strains was also very high in the first three years of the study interval, but in 2025 (25/36; 69.44%) a statistically significant decline was recorded, compared to 2022 (94/102; 92.15%, *p* = 0.001; OR = 5.17; 95% CI:1.87–14.22), with statistical significance maintained even after the Holm correction (*p* = 0.003).

The lowest resistance rate was recorded for colistin. Although the number of resistant strains was relatively stable, a worrying aspect is related to the fact that, in 2025, the percentage of resistant strains to colistin of those tested was more than five times higher *(*16.13%—5/31*)* than in 2022 (3.06%—3/98, *p* = 0.02), but the adjusted *p*-value is not statistically significant (Figure 2, Table S3). Between 2022 and 2025, the data showed a statistically significant downward trend in ceftazidime resistance among *Acinetobacter baumannii* isolates (χ^2^ = 4.54, *p* = 0.033), cefotaxime (χ^2^ = 4.74, *p* = 0.029), ceftriaxone (χ^2^ = 10.48, *p* = 0.001), and meropenem (χ^2^ = 4.466, *p* = 0.034). A significant increasing trend was found only in the case of colistin resistance rate (χ^2^ = 4.334, *p* = 0.03).

#### 3.2.2. Enterobacterales

*E. coli*

Overall, *E. coli* ranked third in frequency, accounting for about 18% (274/1529) of the total Gram-negative pathogens (Figure 1), with a constant and important increase in percentage between 2022 (55/362—15.19%) and 2025 (77/368—20.92%, *p* = 0.05). The highest resistance rate was observed in the case of the third-generation cephalosporins (ceftazidime: 70/251—27.89%; ceftriaxone: 68/254—26.77%) and gentamicin (52/255—20.39%). As for the resistance to carbapenems, it was around 9% (for imipenem—8.22% (18/219), and for meropenem—9.50% (25/263)) (Table S4).

**Figure 3:**
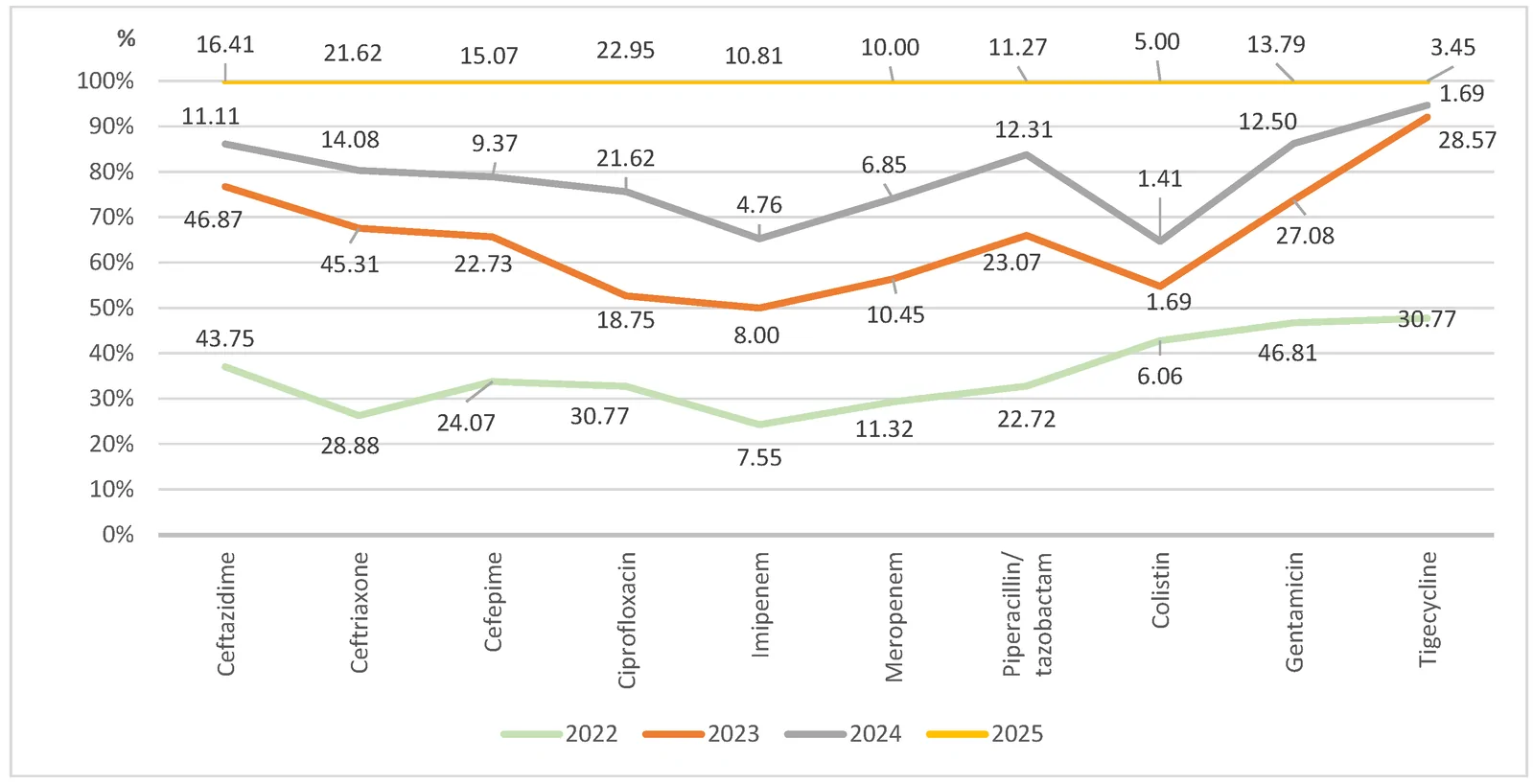
Antimicrobial resistance profile of *E. coli* isolated from endotracheal aspirates, 2022–2025.

By analyzing the trend of the resistance rate of the *E. coli* strains during the studied period, we noticed that, in 2024, a substantial decline in resistance rates was observed for most antibiotics compared with the previous year. In 2025, the study highlighted the resumption of the increase in the percentages of resistant strains, but without statistical significance (Figure 3, Table S4). Thus, in the case of third-generation cephalosporins, a statistically significant decreasing trend was highlighted between 2022 and 2025 among *E. coli* isolates for ceftazidime (χ^2^ = 21.99, *p* < 0.001) and ceftriaxone (χ^2^ = 6.57, *p* = 0.01). The carbapenem-resistance rate was approximately 10% in 2025, marking an increase compared to 2022 in the case of imipenem (7.55%) and a reduction in the case of meropenem (11.32%), but without statistical significance (Figure 3, Table S4).

The lowest resistance was found against colistin, only one strain being resistant in 2023–2024 and two in 2022 and 2025 (Table S4).

*Klebsiella pneumoniae*

**Figure 4:**
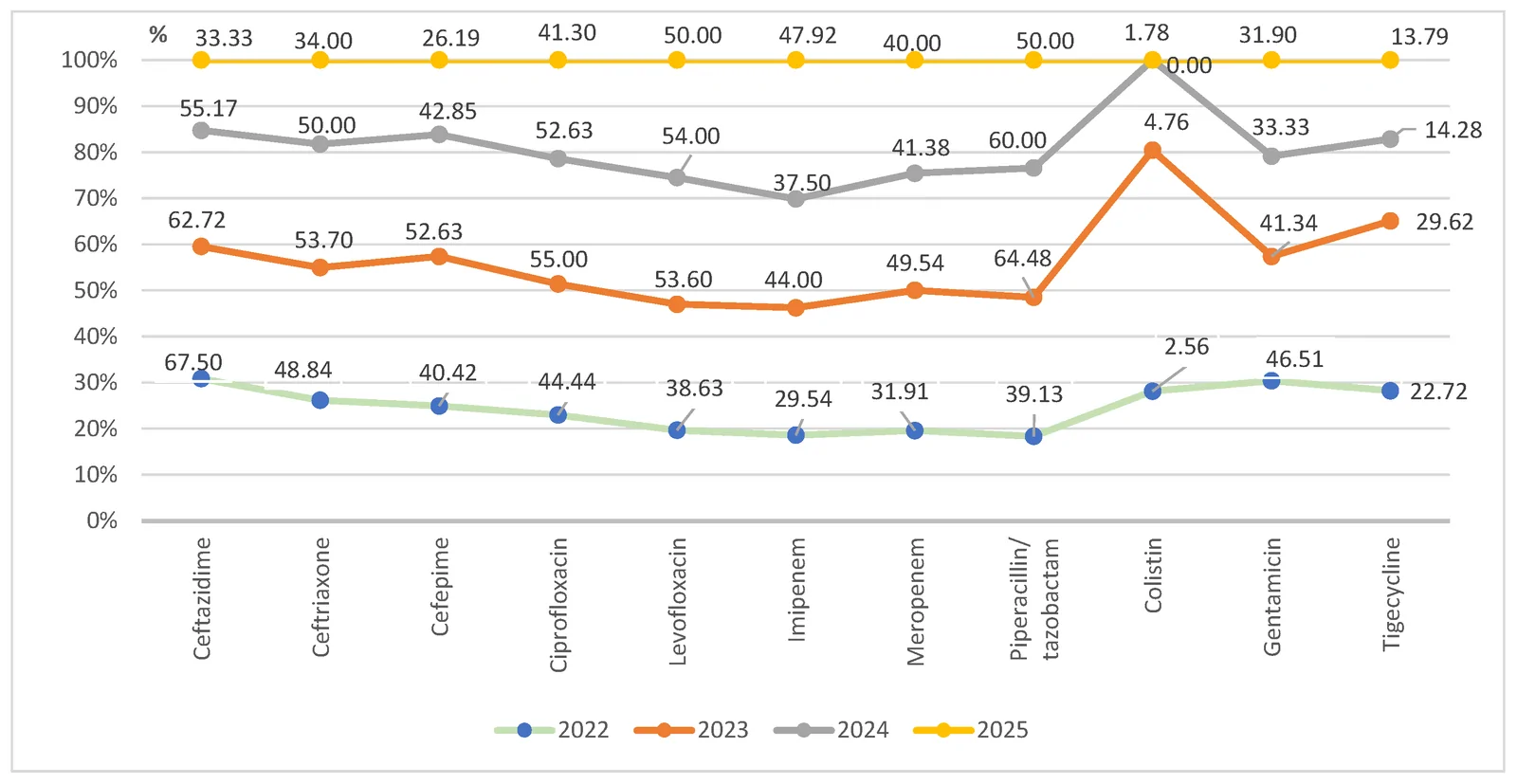
Antimicrobial resistance profile of *Klebsiella pneumoniae* isolated from endotracheal aspirates, 2022–2025.

*Klebsiella pneumoniae* was the fourth most common isolated pathogen, holding almost the same percentage of all samples collected (17,73%—271/1529) as *E. coli*. The analysis of the percentages of the *Klebsiella pneumoniae* strains identified throughout the entire study period showed that the highest resistance was recorded to third-generation cephalosporins (ceftazidime—56.8% (142/250), ceftriaxone—50.20% (124/247)) and to piperacillin–tazobactam (138/246—56.09%). Half of the tested strains were resistant to fluoroquinolones (ciprofloxacin—50% (124/248), respective levofloxacin—50.23% (110/219)) and about 43% to fourth-generation cephalosporins. Around 40% of the *Klebsiella pneumoniae* strains were found to be resistant to carbapenems (imipenem—39.39% (65/165), respective meropenem—42.86% (111/259)) (Figure 4, Table S5).

In 2023, the research highlighted a significant increase in the resistance rates against piperacillin–tazobactam (69/107; 64.48%) compared to the previous year (18/46; 39.13%, *p* = 0.006). In the following years there was a slight decline.

From 2022 to 2025, it was found that the resistance of the tested strains to carbapenems and levofloxacin increased, but without statistical significance. Significant changes were registered in the resistance rate against third-generation cephalosporines. Thus, a significant decreasing trend was found in the case of ceftazidime (χ^2^ = 11.95, *p* < 0.001), the value of the indicator being halved in 2025 (33.33%, 14/42), compared to 2022 (67.5%, 27/40**,** *p* = 0.004; 95% CI:1.65–10.44) (Figure 4, Table S5), difference that remained significant even after the application of the Holm correction (*p* = 0.016). The same trend was observed in the case of ceftriaxone (χ^2^ for trend = 13.45, *p* < 0.001).

Colistin showed the lowest resistance rate, and in 2025 no resistant strain was identified. No statistically significant differences were identified for the other tested antibiotics.

*Proteus mirabilis*

*Proteus mirabilis* isolates represented 5.10% (78/1529) of all the Gram-negative pathogens. Around half of the tested strains were resistant to third-generation cephalosporins (ceftazidime—40/74; 54.05%, respective ceftriaxone—35/71; 49.29%), fluoroquinolones (ciprofloxacin—37/72; 52.77%, levofloxacin—27/55; 49.09%), and gentamicin (37/66; 56.06%). Almost half of the tested strains were resistant to imipenem (29/62–46.77%), while only 17.10% (13/76) were resistant to meropenem (Table S2).

*Enterobacter* spp.

Almost 4% (61/1529) of the identified strains were represented by *Enterobacter* spp. More than a third of these were resistant to third-generation cephalosporins (ceftazidime—21/58; 36.20%, ceftriaxone—22/56; 39.28%), and between 25% (meropenem—15/60) and 30% (imipenem—16/54) were resistant to carbapenems (Table S2).

*Citrobacter* spp.

Approximately 40% of the *Citrobacter* spp. strains tested were resistant to third-generation cephalosporins (ceftazidime—15/37; 40.54%, ceftriaxone—16/38; 42.10%). More than a quarter of the strains were carbapenem-resistant (imipenem—7/26; 26.92%, meropenem—11/39; 28.20%) (Table S2).

*Serratia marcescens*

Around 60% of the tested *Serratia marcescens* strains were resistant to third-generation cephalosporins (ceftazidime—11/18; 61.11%, ceftriaxone—10/17; 58.82%). Two-thirds of the tested strains were resistant to imipenem (10/15), while almost 40% (7/18) were resistant to meropenem.

*Providencia* spp.

Although the study identified a small number of strains of *Providencia* spp. (16), these showed more than 80% resistance to third-generation cephalosporins (ceftazidime—13/16; 81.25%, ceftriaxone—12/14; 85.71%) and imipenem (11/13; 84.61%). Over 60% of the strains (10/16) were resistant to meropenem (Table S2).

#### 3.2.3. *Pseudomonas aeruginosa*

*Pseudomonas aeruginosa* was the most frequently isolated pathogen in the endotracheal aspirates collected from patients admitted to the ICU, accounting for approximately 28% (422/1529) of the total detected Gram-negative pathogens. During 2022–2025, the highest resistance was identified against imipenem (235/381; 61.68%) and ceftazidime (241/401; 60.10%), in the case of the other antibiotics tested, the resistance being around 50% (Table S2).

**Figure 5:**
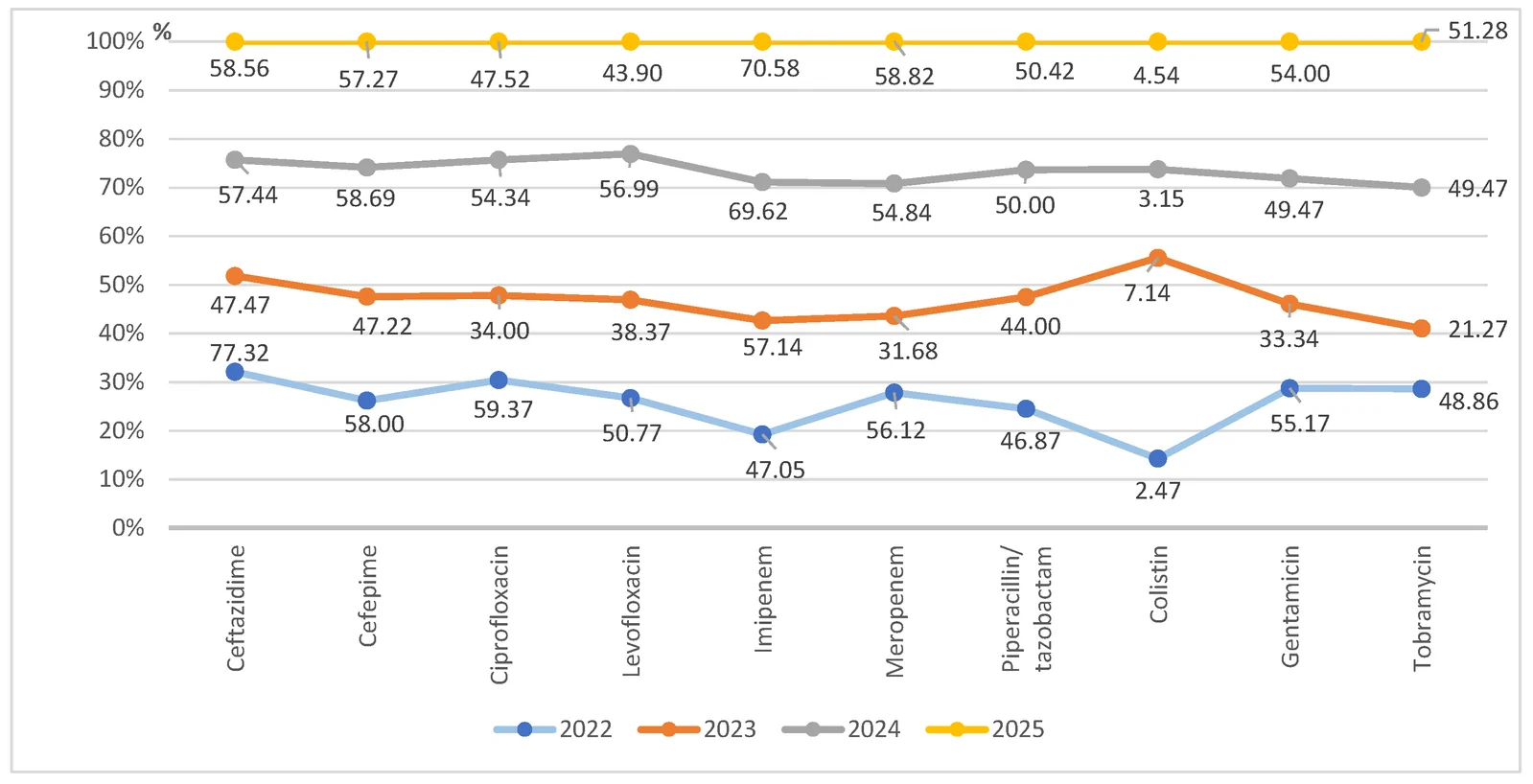
Antimicrobial resistance profile of *Pseudomonas aeruginosa* isolated from endotracheal aspirates, 2022–2025.

The antimicrobial resistance profile of *Pseudomonas aeruginosa* demonstrated statistically significant changes in resistance rates during the study period. Imipenem resistance demonstrated a statistically significant increasing trend throughout the entire study period (χ^2^ for linear trend = 20.74, *p* < 0.001), rising from 47.05% (40/85) in 2022 to 70.58% (84/119) in 2025 (*p* = 0.001; OR = 0.37; 95% CI:0.20–0.66) (Figure 5, Table S6). After applying the Holm correction, the difference remains statistically significant, with adjusted *p* = 0.004.

A significant decrease in resistance rate from 2022 (75/97; 77.32%) to 2025 (65/111; 58.56%, *p* = 0.006; OR = 2.41; 95% CI:1.31–4.42) was highlighted for ceftazidime (Figure 5, Table S6), the difference between 2022 and 2025 remaining statistically significant (*p* = 0.018) after the application of the Holm correction. Colistin maintained the lowest resistance rates throughout the study period, the highest value being observed in 2023 (7.14%—7/98), with no statistically significant differences between years. These results confirm the maintenance of the efficacy of this last-resort antibiotic (Figure 5).

#### 3.2.4. Multidrug Resistance of the Gram-Negative Pathogens

This research evaluated also the annual distribution of resistance categories, specifically MDR, XDR, PDR, and non-resistant isolates, in order to identify the temporal trends and highlight possible significant changes in the resistance patterns over time.

**Figure 6:**
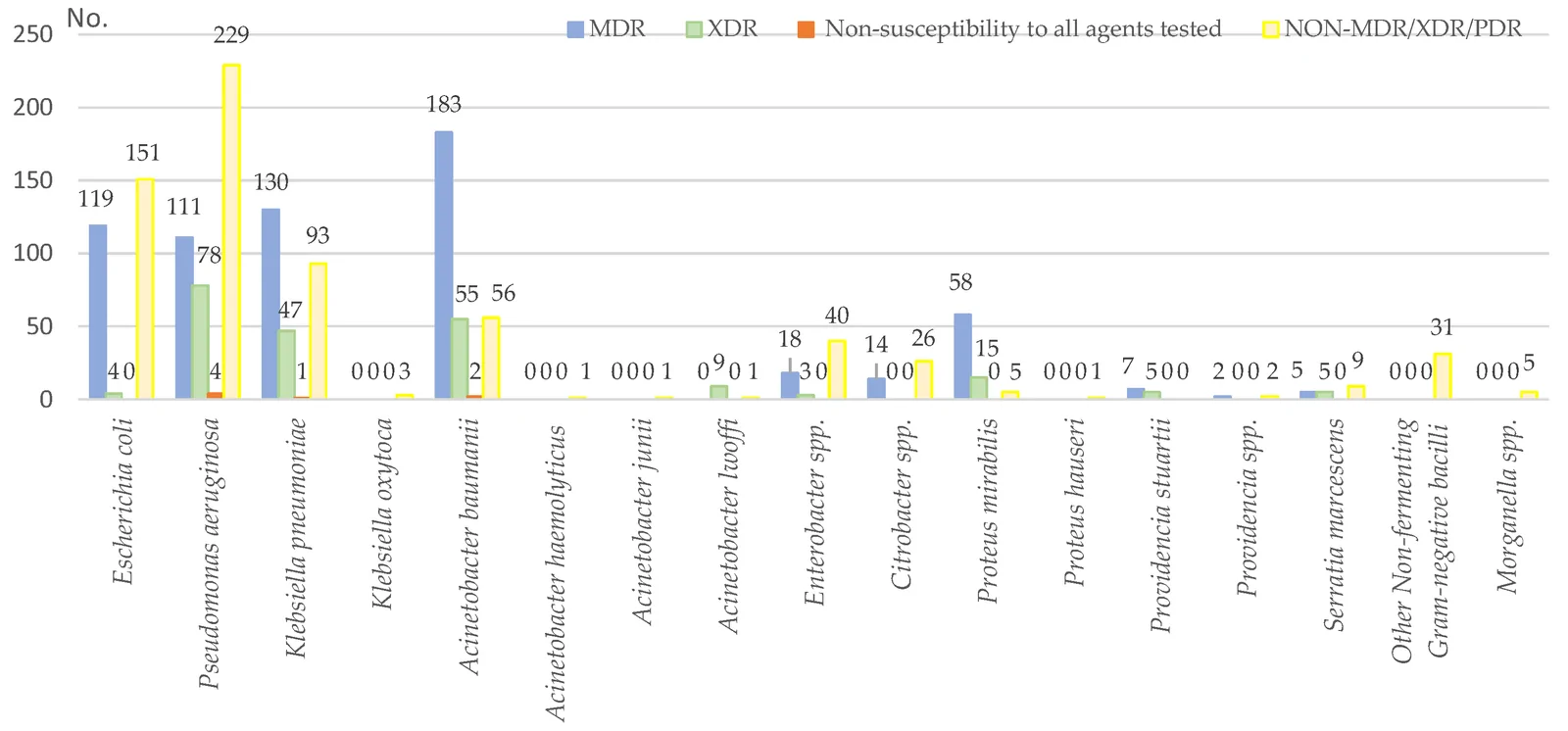
Antimicrobial resistance classification of all Gram-negative microorganisms isolated from endotracheal aspirates 2022–2025; MDR—multidrug-resistant; XDR—extensively drug-resistant; PDR—pan-drug-resistant. MDR, XDR and non-susceptibility to all agents’ status were recorded for each bacterial isolate; NON-MDR/XDR/PDR—without MDR/XDR/PDR criteria.

The data revealed that over 40% of all Gram-negative strains isolated were MDR (647/1529; 42.31%) and almost 15% were XDR (221/1529; 14.45%). Seven strains (0.46%) were not-susceptible to all agents tested, of which 4 were *Pseudomonas aeruginosa* (Figure 6).

**Figure 7:**
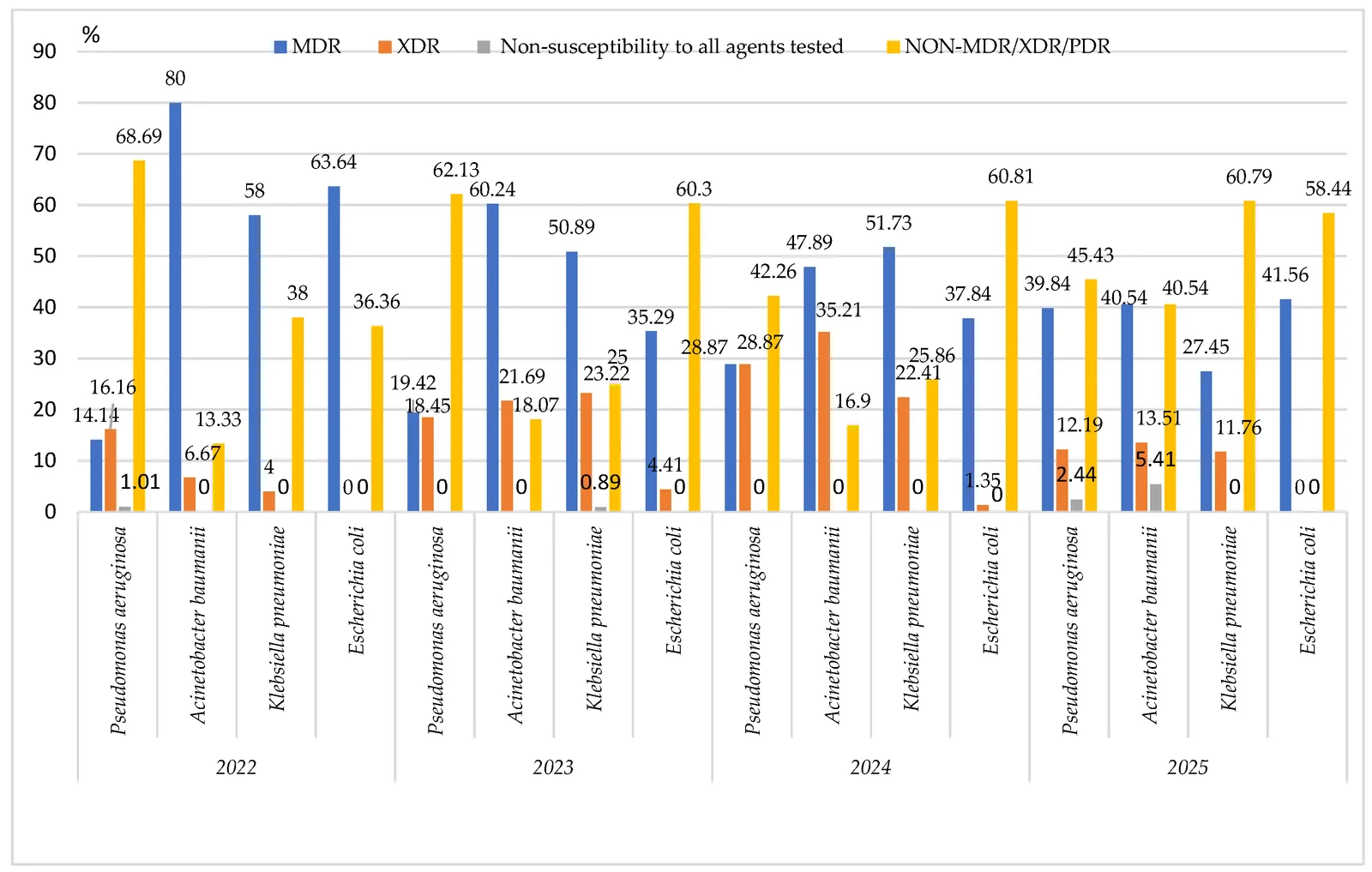
Antimicrobial resistance classification of the critical and high priority Gram-negative microorganisms isolated from endotracheal aspirates, 2022–2025; MDR—multidrug-resistant; XDR—extensively drug-resistant;. NON-MDR/XDR/PDR—without MDR/XDR/PDR criteria.

Among the isolated Gram-negative bacteria, the most frequent MDR species was *Acinetobacter baumannii*, accounting for almost 30% of all the MDR Gram-negative strains (183/647) (Figure 6). Approximately 60% (183/296) of all *Acinetobacter baumannii* strains were MDR and almost 19% were XDR (55/296) (Figure 7). The analysis of the distribution of resistance profiles across the years demonstrated a significant decreasing trend in the proportions of MDR strains (χ^2^ = 26.51, *p* <0.001), from 80% (84/105) in 2022 to 40.54% (15/37) in 2025 (Figure 7). After the application of the Holm correction, the difference remained significant (*p* = 0.00004). In contrast, a significant increasing trend was recorded for the percentage of XDR isolates (χ^2^ = 8.74, *p* = 0.003), which significantly increased between 2022 (7/105; 6.67%) and 2023 (18/83; 21.69%, *p* = 0.004), reaching its maximum value in 2024 (25/71; 35.31%). The difference in XDR rates between 2022 and 2023 remained statistically significant after applying the Holm correction (Holm-adjusted *p* = 0.016). In 2025, there was a marked decrease in the number of identified XDR strains (5 cases), but their percentage, given the small number of isolated strains, was twice as high as at the beginning of the interval. Also, in the same year, two strains not-susceptible to all agents tested were identified, which can also indicate possible changes in the resistance profile of this pathogen (Figure 7). These strains were resistant to the following antibiotics: amikacin, aztreonam, gentamicin, tobramycin, imipenem, meropenem, ciprofloxacin, levofloxacin, cefotaxime, ceftazidime, ceftriaxone, cefepime, piperacillin–tazobactam, tigecycline, and colistin.

Almost half of all the *Klebsiella pneumoniae* strains were MDR (130/271; 47.97%), representing 20% of all the MDR Gram-negative strains (130/647) (Figure 6). Around 17% were XDR (47/271) (Figure 6). The percentage of MDR strains remained high in 2022 (29/50; 58%), 2023 (57/112; 50.89%) and 2024 (30/58; 51.73%), but in 2025 was reduced by almost half (14/51; 27.45%, *p* < 0.001), a significant decreasing trend being registered during the four-year period (χ^2^ = 8.43, *p* = 0.003). After the application of the Holm correction, the significant difference between 2022 and 2025 (adjusted *p* = 0.012) and between 2024 and 2025 (adjusted *p* = 0.030) was also confirmed. Important changes were observed in the case of XDR strains, the maximum number being registered in 2023 (26/112; 23.22%), significantly higher (*p* < 0.001) compared to 2022 (2/50;4%). A similar percentage was recorded in 2024 (13/58; 22.41%), but this was halved in 2025 (6/51; 11.76%). One strain identified in 2023 showed non-susceptibility to all agents tested (Figure 7), as follows: ampicillin, amoxicillin/clavulanic acid, aztreonam, chloramphenicol, ticarcillin, piperacillin–tazobactam, cefoxitin, ceftazidime, ceftriaxone, cefepime, ertapenem, imipenem, meropenem, amikacin, gentamicin, tobramycin, ciprofloxacin, levofloxacin, moxifloxacin, ofloxacin, tigecycline, and colistin.

More than 25% of all the *Pseudomonas aeruginosa* strains (111/422) were MDR (Figure 6), holding a percentage of 17.16% of the total MDR Gram-negative strains, with a constant and significant increasing trend (χ^2^ = 21.51, *p* <0.001) during the study period. The percentage of MDR *Pseudomonas aeruginosa* strains was almost three times higher in 2025 (39.84%; 49/123) compared to 2022 (14.14%; 14/99, *p* < 0.001). The Holm-adjusted analysis demonstrates also a significant difference between the beginning and end of the study period (adjusted *p* = 0.00016).

The number of XDR isolates has steadily grown, from 16 cases in 2022 (16.16%) to 28 in 2024 (28.87%). In 2025 a significant reduction was recorded, with 15 XDR strains being identified (12.19%, *p* = 0.003). Following Holm correction for multiple comparisons, the statistically significant difference between 2024 and 2025 was maintained (adjusted *p* = 0.012). However, in the same year, three of the four *Pseudomonas aeruginosa* strains that were non-susceptible to all tested antimicrobial agents during the entire study period were identified. This fact can indicate a possible change in the resistance profile of *Pseudomonas aeruginosa* (Figure 7). The four strains were non-susceptible to the following tested antimicrobial agents: ampicillin-sulbactam, amikacin, tobramycin, ciprofloxacin, levofloxacin, piperacillin–tazobactam, cefoxitin, cefuroxime, ceftazidime, ceftriaxone, cefepime, imipenem, meropenem, aztreonam, tetracycline, tigecycline, and colistin.

The study also found that more than 40% of *E. coli* strains were MDR (119/274). Most of them were isolated in 2022 (35/55; 63.64%), after which their prevalence decreased significantly (*p* = 0.02), reaching 41.56% (32/77) in 2025. Three XDR *E. coli* strains were found in 2023 and one in 2024. No strain non-susceptible to all the tested agents was found.

Almost 75% of the *Proteus mirabilis* strains were MDR (58/78) and about 20% were XDR (Figure 6).

## 4. Discussion

The present research is a four-year retrospective study, providing an assessment of the distribution and antimicrobial resistance patterns in critical and high-priority Gram-negative isolates from endotracheal aspirates collected from ICU patients at a tertiary care center in Romania between 2022 and 2025.

*Pseudomonas aeruginosa* was the most frequently isolated Gram-negative microorganism (27.60%), followed by *Acinetobacter baumannii* (19.36%), *E. coli* (17.92%), and *Klebsiella pneumoniae* (17.73%).

In other similar studies, the same predominant Gram-negative pathogens were identified in endotracheal aspirates. Thus, *Klebsiella* spp., *Acinetobacter* spp., and *Pseudomonas* spp. have been isolated as a priority in a study conducted in a tertiary hospital in Bangladesh, from June 2023 to July 2024 [25], as well as in the study carried out in Turkey, between 2018 and 2022 [26]. *Klebsiella pneumoniae* was the most frequent specimen type isolated from tracheal aspirate in another study conducted in a tertiary Romanian ICU between 2021 and 2024 [27]. The predominance of highly resistant Gram-negative pathogens in the endotracheal aspirates collected from ICU patients was also highlighted in the study conducted in 2025 in Romania by Miron et al. [5], *Acinetobacter baumannii* and *Pseudomonas aeruginosa* being the most frequently isolated pathogens. In research conducted in Italy by Biagio Santella et al. between 2015 and 2019, the most frequent were isolated *Acinetobacter baumannii*, *Pseudomonas aeruginosa* and *Klebsiella pneumoniae* [28], while *Pseudomonas* spp., *Klebsiella pneumoniae,* and *Acinetobacter* spp. were most commonly identified between 2018 and 2019 in the study developed by Shambhavi Singh et al. [29]

Our study revealed a significant increase in the percentage of *Pseudomonas aeruginosa*, while a significant decline was observed for *Acinetobacter baumannii.* The data revealed a high proportion of the Gram-negative MDR and XDR strains, and seven strains non-susceptible to all the tested agents were also identified, in accordance with the surveillance methodology for healthcare-associated infections and antimicrobial resistance, developed by the Romanian Ministry of Health [22].

The results indicate a significant upward trend in carbapenem resistance among *Pseudomonas aeruginosa* isolates, reaching nearly 60% for meropenem and exceeding 70% for imipenem in 2025. Furthermore, the percentage of MDR *Pseudomonas aeruginosa* strains increased considerably throughout the study period, almost tripling in 2025 compared to 2022, a year in which almost 40% of the strains tested were MDR. In contrast, a significant decline was registered in 2025 compared to 2024 for XDR isolates, around 12% of the strains having this resistance profile. However, four of the seven strains non-susceptible to all the tested agents identified throughout the study period were *Pseudomonas aeruginosa*, and three of them were detected in 2025, suggesting a trend of difficult-to-treat strain selection. These changes may reflect variations in antimicrobial exposure and prescribing practices, selective pressure associated with prolonged ICU stays and mechanical ventilation, dissemination of resistant strains, or changes in infection-control practices and patient characteristics.

These findings have important clinical consequences, considering the fact that the increasing proportion of carbapenem-resistant and MDR isolates is particularly alarming, as it substantially limits therapeutic options and complicates the management of severe infections, especially in critically ill and immunocompromised patients with lower respiratory tract infections. These may lead to higher treatment costs, increased rates of treatment failure, and poorer clinical outcomes, including increased mortality [30]. These observations emphasize the necessity of sustained and coordinated interventions, including antimicrobial stewardship programs and regular review of empirical treatment protocols based on the resistance of the strains circulating in the hospital; prompt de-escalation of therapy once microbiological results become available; and exploring the use of other therapeutic agents that have been shown to be effective in *Pseudomonas* MDR infections [31].

*Acinetobacter baumannii* showed the highest resistance levels among all Gram-negative pathogens included in this study. The data highlighted a statistically significant downward trend in resistance to third-generation cephalosporins and carbapenem resistance. This reduction should be interpreted with caution, taking into account the fact that in 2025 the number of strains of *Acinetobacter baumannii* was approximately half compared to the previous year. These results need to be the subject of future research to confirm if the trend continues.

A halving of MDR *Acinetobacter baumannii* strains has been observed between 2022 and 2025, which may suggest the positive impact of infection prevention and control measures and the adoption of a judicious antibiotic administration policy. However, this decrease in MDR was accompanied by a significant increase in the prevalence of XDR isolates between 2022 and 2024, exceeding 35%. Although only five XDR strains were identified in 2025, their prevalence was twice as high as in 2022, with two strains non-susceptible to all the tested agents also being identified. This finding can reflect a possible change in the resistance profile of *Acinetobacter baumannii* strains, with the selection of XDR and strains non-susceptible to all the tested agents, rather than indicating a true reduction in the burden of antimicrobial resistance. This fact can be explained by the prolonged duration of administration of antibiotic therapy to critically ill ICU patients and also by the excessive empirical administration of antibiotics, including reserve antibiotics, prior to the release of the results of the microbiological examinations. This development draws attention to the possibility of selecting critical pathogens resistant to most of the available antibiotics, changing from MDR to XDR or even PDR.

These observed trends raise serious concern regarding the existing therapeutic options for these critical patients, taking into account that *Acinetobacter baumannii* is involved in the etiology of approximately 20% of the infections reported in intensive care units, ranking among the most common Gram-negative pathogens responsible for hospital-acquired infections worldwide [32]. Several other studies have also shown a high percentage of MDR strains of A*cinetobacter* spp. and alarming rates of resistance levels to carbapenems and third-generation cephalosporins [5,33,34,35].

Our study showed the lowest resistance rates against colistin overall. However, a worrying aspect is related to the fact that in the period 2022–2025, the percentage of resistant strains to colistin among those tested was five times higher, with a significant increasing trend in the resistance rate. This finding draws attention to the use of this reserve antibiotic with caution. Its use should be restricted to situations in which susceptibility data and clinical indications clearly justify administration, for example, as a last-resort therapeutic option for pneumonia caused by carbapenem-resistant *A. baumannii* (CRAB), especially in critically ill patients with ventilator-associated pneumonia (VAP) [15]. Even in these situations, the administration of this last-line antibiotic should be done with caution, given that its clinical use is limited by dose-dependent nephrotoxicity and neurotoxicity [36]. In the case of critically ill patients who exhibit complex pharmacokinetics for colistin, individual dose adjustments are required to avoid toxicity and achieve therapeutic efficacy [37].

Although most studies report a low rate of resistance to colistin [28,33,34], which remains the most effective antibiotic, a systematic review published in 2024 highlighted that resistance to colistin among *A. baumannii* isolates has tripled from 3.2% pre-2015 to 8.9% post-2020, particularly in ICU environments [38]. This finding draws attention to the threat of selecting colistin-resistant strains and underscores the need to discover new antibiotics for treating MDR/XDR Gram-negative infections. Consideration should also be given to optimizing therapeutic strategies, including the use of evidence-based combination regimens when appropriate [15], while ongoing research into novel antimicrobial agents and alternative treatment approaches remains crucial to address the diminishing effectiveness of this critical last-line antibiotic.

In our research, over the four-year period, the data demonstrate a significant decreasing trend in the antimicrobial resistance of *Klebsiella pneumoniae* against third-generation cephalosporines. On the other hand, the resistance of the tested strains to carbapenems increased between 2022 and 2025, especially for imipenem, reaching almost 48%. These results were consistent with other findings [38,39,40,41,42].

The progressive increase in carbapenem resistance observed in the present study is particularly concerning, given that it represents a major cause of high-mortality hospital-related infections [43]. The results are in line with those reported at the national level, Romania being one of the Eastern European countries with the highest number of carbapenem-resistant Enterobacterales cases, along with Greece, Poland, and Serbia [44]. This situation represents a major clinical concern, as these drugs are typically used as last-line methods of treatment for severe infections due to MDR Gram-negative bacteria. These results draw attention to the fact that the increased proportion of carbapenem-resistant strains is an important health problem, identified as such globally by the introduction by the World Health Organization of carbapenem-producing Enterobacterales on the list of critical priority pathogens [13].

Data from our study revealed that around 48% of all the *Klebsiella pneumoniae* strains were MDR. However, a significant reduction was recorded in 2025, reaching approximately 27%. The same trend was observed in the case of XDR strains, with their prevalence halving compared to the value recorded in 2023 and 2024. The results are consistent with those reported by Sava et al. [27], who observed a decrease in MDR among *Klebsiella pneumoniae* isolates from ICU patients. However, in this study, an increasing trend in the proportion of XDR and even PDR phenotypes was observed during 2023 and 2024. A similar trend was identified in our research for *Acinetobacter baumannii*. The study conducted between 2014 and 2016, in Palermo, Italy [45], revealed the presence of a high-risk carbapenem-producing *Klebsiella pneumoniae* clone (ST101) and a resistant strain to colistin belonging to ST405, drawing attention on the important changes in the dissemination and genetic characteristics of *Klebsiella pneumoniae* in this region of Italy. These data are consistent with those reported at the national level, in accordance with which more than half of the invasive strains of *Acinetobacter* spp. and *Klebsiella pneumoniae* were found to be resistant to carbapenems (imipenem/meropenem) [46], antibiotics classified by WHO as critically important antimicrobials for human medicine [47], and considered the most effective last-line therapeutic agents for treating infections caused by multidrug-resistant Gram-negative pathogens [48].

According to the results of our study, during the four-year period, *E. coli* demonstrated a significant decreasing trend in the resistance rate for third-generation cephalosporines.

Around 10% of the *E. coli* strains were found to be resistant to carbapenems in 2025, with an increasing trend compared to 2022 for imipenem and a reduction in the case of meropenem, but without statistical significance. Although decreasing in the studied interval, the percentage of MDR *E. coli* remained high, exceeding 40% in 2025. The high carbapenem-resistance rates observed in this study are especially concerning because these antimicrobial classes are frequently used in our hospital for the treatment of critically ill patients.

The identification of strains non-susceptible to all the tested agents, even in very small numbers, draws attention to the need to implement sustained preventive and control measures and the need for judicious administration of antibiotics.

Although the fact that the lowest resistance rate was recorded against colistin indicates the maintenance of this therapeutic possibility for cases of severe infections in critically ill patients, excessive dependence on last-line agents may accelerate the emergence of pan-resistant strains.

Implementation of antimicrobial stewardship programs, optimization of empirical therapy based on local resistance patterns and antimicrobial consumption data, rapid diagnostic methods, using new diagnostic technologies [49], and strict infection prevention strategies remain essential measures to limit the transmission of multidrug-resistant Gram-negative bacteria in hospital patients [50].

The findings of the present study indicate the necessity of continuous microbiological surveillance of antimicrobial resistance, especially in intensive care units, to rapidly identify emerging resistance trends and adapt empirical treatment guidelines; strengthening of infection prevention and control measures, including hand hygiene, standard precautions, environmental decontamination, ventilator-associated pneumonia prevention, and screening of ICU patients; isolation of the infected/colonized patients with MDR/XDR/PDR pathogens; and periodic reevaluation of hospital antibiotic policies based on local resistance epidemiology.

Our study has certain limitations that need to be acknowledged. The research is a retrospective analysis of microbiological data collected from ICU patients admitted to a single tertiary hospital, which may limit the generalizability of the findings with different categories of patients, other antimicrobial prescribing patterns, and infection control practices. Another limitation is the lack of complete clinical outcome data, such as ICU mortality, ICU length of stay, or duration of mechanical ventilation with no possibility of correlating the results of the microbiological examination with the clinical and radiological aspects, and also the fact that ESBL or carbapenemase production was not phenotypically or molecularly confirmed.

However, the study provides valuable information based on the latest regarding antimicrobial resistance, including the percentage of the MDR and XDR critical and high-priority Gram-negative pathogens isolated from endotracheal aspirates in ICU patients admitted to one of the largest hospitals in Romania. The research includes a temporal trend analysis over an extended period of four years, which can help identify and evaluate resistance patterns over time, serving as a basis for updating the infection prevention and control programs and antibiotic administration protocols at the hospital. Future multicenter studies with integrated molecular surveillance can contribute to supporting and generalizing the results of this study and improving treatment outcomes for critically ill patients with Gram-negative lower respiratory tract infections.

## 5. Conclusions

This study highlighted a significant decline in the proportions of MDR isolates of *Klebsiella pneumoniae* and *Acinetobacter baumannii* among endotracheal aspirates in ICU patients and a significant increasing trend for the percentage of XDR *Acinetobacter baumannii* isolates and MDR *Pseudomonas aeruginosa* strains.

These findings underscore the role of continuous microbiological and epidemiological surveillance and the need for the implementation of strategies for reducing the spread of MDR respiratory pathogens: strengthening local resistance surveillance, regular updating of empirical treatment protocols, and reinforcement of infection prevention and control measures.

## Figures and Tables

**Table 2:** Distribution of the endotracheal aspirates in which Gram-negative pathogens have been identified by gender and age group.

*Patients*		*Year*				
		2022	2023	2024	2025	Total
Gender	Male	190 (59%)	250 (66.31%)	214 (61.32%)	207 (60.35%)	861 (61.90%)
	Female	132 (41%)	127 (33.69%)	135 (38.68%)	136 (39.65%)	530 (38.10%)
Total		322	377	349	343	1391
Age	18–64 years	119 (36.96%)	114 (30.24%)	91 (26.07%)	83 (24.20%)	407 (29.26%)
	≥65 years	203 (63.04%)	263 (69.76%)	258 (73.93%)	260 (75.80%)	984 (70.74%)
Total		322	377	349	343	1391

## Data Availability

The original contributions presented in this study are included in the article/supplementary material. Further inquiries can be directed to the corresponding author.

## Supplementary Materials

The following supporting information can be downloaded at https://www.mdpi.com/article/10.3390/microorganisms14102281/s1: Table S1: Distribution of Gram-negative pathogens isolated from endotracheal aspirates, 2022–2025; Table S2. Antimicrobial resistance pattern of the main critical and high priority Gram-negative pathogens isolated from endotracheal aspirates, 2022–2025; Table S3. Antimicrobial resistance pattern of the *Acinetobacter baumannii* isolated from endotracheal aspirates, 2022–2025; Table S4. Antimicrobial resistance pattern of the *E. coli* isolated from endotracheal aspirates, 2022–2025; Table S5. Antimicrobial resistance pattern of the *Klebsiella pneumoniae* isolated from endotracheal aspirates, 2022–2025; Table S6. Antimicrobial resistance pattern of the *Pseudomonas aeruginosa* isolated from endotracheal aspirates, 2022–2025.
