# Supplementary material for: Distribution and Antimicrobial Resistance Patterns of Critical and High-Priority Gram-Negative Pathogens Isolated from Endotracheal Aspirates in an Intensive Care Unit
Source: Microorganisms. 2026 Oct 7;14(10):2281. doi: 10.3390/microorganisms14102281 (PMC13707979; doi:10.3390/microorganisms14102281)
Supplement: Supplementary file 1 [file microorganisms-14-02281-s001.zip › Table S1.pdf]

Table S1. Distribution of Gram-negative pathogens isolated from endotracheal aspirates, 2022-2025

Pathogen	Year				2022-2025 (N=1529)
	**2022** (N=362)	2023 (N=425)	**2024** (N=374)	**2025** (N=368)	
	**N=, (%)**	**N=, (%)**	**N=, (%)**	**N=, (%)**	**N=, (%)**
Pseudomonas aeruginosa	99 (27.35)	103 (24.24)	97 (25.94)	123 (33.42)	422 (27.60)
Acinetobacter baumannii	105 (29)	83 (19.53)	71 (18.98)	37 (10.06)	296 (19.36)
Acinetobacter lwoffii	4 (1.10)	4 (0.94)	1 (0.27)	1 (0.27)	10 (0.65)
Acinetobacter junii	0 (0)	1 (0.23)	0 (0)	0 (0)	1 (0.06)
Acinetobacter haemolyticus	0 (0)	0 (0)	1 (0.27)	0 (0)	1 (0.06)
Klebsiella pneumoniae	50 (13.81)	112 (26.35)	58 (15.51)	51 (13.86)	271 (17.73)
Klebsiella oxytoca	1 (0.28)	1 (0.24)	1 (0.27)	0 (0)	3 (0.20)
Escherichia coli	55 (15.19)	68 (16)	74 (19.79)	77 (20.92)	274 (17.92)
Proteus mirabilis	21 (5.80)	26 (6.12)	17 (4.54)	14 (3.81)	78 (5.10)
Proteus hauseri.	1 (0.28)	0 (0)	0 (0)	0 (0)	1 (0.06)
Other Non-fermenting Gram-negative bacilli	15 (4.14)	9 (2.12)	2 (0.53)	5 (1.36)	31 (2.03)
Citrobacter sp.	1 (0.28)	7 (1.65)	15 (4.01)	17 (4.62)	40 (2.62)
Enterobacter sp.	6 (1.66)	5 (1.18)	22 (5.88)	28 (7.61)	61 (3.99)
Providencia stuartii	1 (0.28)	3 (0.71)	4 (1.07)	4 (1.09)	12 (0.79)
Providencia spp.	1 (0.28)	1 (0.23)	1 (0.27)	1 (0.27)	4 (0.26)
Serratia marcescens	2 (0.55)	1 (0.23)	8 (2.14)	8 (2.17)	19 (1.24)
Morganella spp.	0 (0)	1 (0.23)	2 (0.53)	2 (0.54)	5 (0.33)
