# Supplementary material for: Distribution and Antimicrobial Resistance Patterns of Critical and High-Priority Gram-Negative Pathogens Isolated from Endotracheal Aspirates in an Intensive Care Unit
Source: Microorganisms. 2026 Oct 7;14(10):2281. doi: 10.3390/microorganisms14102281 (PMC13707979; doi:10.3390/microorganisms14102281)
Supplement: Supplementary file 1 [file microorganisms-14-02281-s001.zip › Table S2.pdf]

Table S2. Antimicrobial resistance pattern of the main critical and high priority Gram-negative pathogens isolated from endotracheal aspirates, 2022-2025

**Antimicrobial agent**	***Acinetobacter baumanii*** (n=296)	***E. coli*** (n=274)	***Klebsiella pneumoniae*** (n=271)	***Pseudomonas aeruginosa*** (n=422)	***Proteus mirabilis*** (n=78)	***Enterobacter spp.*** (n=61)
Ceftazidime	267/289 (92,38%)	70/181 (27.89%)	142/250 (56.8%)	241/401 (60.10%)	40/74 (54.05%)	21/58 (36.20%)
Ceftriaxone	182/199 (91,46%)	68/254 (26.77%)	124/247 (50.20%)	-	35/71 (49.29)	22/56 (39.28%)
Cefotaxime	236/254 (92,91%)	-	-	-	-	-
Cefepime	211/261 (80.84%)	40/235 (17.02%)	91/214 (42.52%)	205/367 (55.85%)	16/62 (25.80%)	15/57 (26.31%)
Imipenem	220/262 (83,97%)	18/219 (8.22%)	65/165 (39.39%)	235/381 (61.68%)	29/62 (46.77%)	16/54 (29.62%)
Meropenem	255/290 (87.93%)	25/263 (9.50%)	111/259 (42.86%)	208/411 (50.61%)	13/76 (17.10%)	15/60 (25%)
Ciprofloxacin	254/285 (89.12%)	58/251 (23.11%)	124/248 (50%)	189/389 (48.58%)	38/72 (52.77%)	15/56(26.78%)
Levofloxacin	210/251 (83.66%)	29/163 (17.79%)	110/219 (50.23%)	137/285 (48.07%)	27/55 (49.09%)	8/37 (21.62%)
Piperacillin/ tazobactam	257/291 (88.31%)	-	138/246 (56.09%)	195/407 (47.91%)	12/73 (16.43%)	12/51 (23.53%)
Colistin	16/277 (5.77%)	6/203 (2.95%)	7/230(3.04%)	15/340 (4.41%)	59/59 (100%)*	3/44 (6.82%)
Gentamicin	238/288 (82.64%)	52/225 (23.11%)	97/251 (38.64%)	183/384 (47.65%)	37/66(56.06%)	14/56 (25%)
Tobramycin	-	-	-	170/394 (43.15%)	-	-
Tigecycline	70/212 (33.01%)	25/190 (13.16%)	26/128 (20.31%)	-	52/54 (96.29%)	1/35 (2.86%)

- not tested; * - Proteus spp. is intrinsically resistant to colistin
