# Supplementary material for: Distribution and Antimicrobial Resistance Patterns of Critical and High-Priority Gram-Negative Pathogens Isolated from Endotracheal Aspirates in an Intensive Care Unit
Source: Microorganisms. 2026 Oct 7;14(10):2281. doi: 10.3390/microorganisms14102281 (PMC13707979; doi:10.3390/microorganisms14102281)
Supplement: Supplementary file 1 [file microorganisms-14-02281-s001.zip › Table S3.pdf]

Table S3. Antimicrobial resistance pattern of the *Acinetobacter baumanii* isolated from endotracheal aspirates, 2022-2025

Antimicrobial agent	**2022** (n=105)	**2023** (n=83)	**2024** (n=71)	**2025** (n=37)
Ceftazidime	99/103 (96.11%)	72/80 (90%)	68/71 (95.77%)	28/35 (80%)
Ceftriaxone	71/74 (95.94%)	43/46 (93.48%)	42/44 (95.45%)	26/35 (74.28%)
Cefotaxime	82/86 (95.34%)	66/72 (91.67%)	61/61 (100%)	27/35 (77.14%)
Cefepime	74/100 (74%)	51/59 (86.44%)	61/66 (92.42%)	25/36 (69.44%)
Imipenem	79/102 (77.45%)	71/79 (89.87%)	53/57 (92.98%)	17/24 (70.83%)
Meropenem	94/102 (92.15%)	69/82 (84.14%)	67/70 (95.71%)	25/36 (69.44%)
Ciprofloxacin	96/103 (93.20%)	67/80 (83.75%)	67/70 (95.71%)	24/32 (75%)
Levofloxacin	69/87 (79.31%)	51/61 (83.60%)	66/69 (95.65%)	24/34 (70.59%)
Piperacillin/ tazobactam	91/103 (88.35%)	70/82 (85.36%)	68/70 (97.14%)	28/36 (77.78%)
Colistin	3/98 (3.06%)	5/79 (6.33%)	3/69 (4.35%)	5/31 (16.13%)
Gentamicin	96/104 (92.30%)	60/78 (76.92%)	58/70 (82.85%)	24/36 (66.67%)
