# Supplementary material for: Distribution and Antimicrobial Resistance Patterns of Critical and High-Priority Gram-Negative Pathogens Isolated from Endotracheal Aspirates in an Intensive Care Unit
Source: Microorganisms. 2026 Oct 7;14(10):2281. doi: 10.3390/microorganisms14102281 (PMC13707979; doi:10.3390/microorganisms14102281)
Supplement: Supplementary file 1 [file microorganisms-14-02281-s001.zip › Table S4.pdf]

Table S4. Antimicrobial resistance pattern of the *E coli* isolated from endotracheal aspirates, 2022-2025

Antimicrobial agent	**2022** (n=55)	**2023** (n=68)	**2024** (n=74)	**2025** (n=77)
Ceftazidime	21/48(43.75%)	30/64 (46.87%)	8/72 (11.11%)	11/67 (16.41%)
Ceftriaxone	13/45 (28.88%)	29/64 (45.31%)	10/71 (14.08%)	16/74 (21.62%)
Cefepime	13/54 (24.07%)	10/44 (22.73%)	6/64 (9.37%)	11/73 (15.07%)
Imipenem	4/53 (7.55%)	4/50 (8%)	2/42 (4.76%)	8/74 (10.81%)
Meropenem	6/53 (11.32%)	7/67 (10.45%)	5/73 (6.85%)	7/70 (10%)
Ciprofloxacin	16/52 (30.77%)	12/64 (18.75%)	16/74 (21.62%)	14/61 (22.95%)
Piperacillin/ tazobactam	10/44 (22.72%)	15/65 (23.07%)	8/65 (12.31%)	8/71 (11.27%)
Colistin	2/33 (6.06%)	1/59 (1.69%)	1/71 (1.41%)	2/40 (5%)
Gentamicin	22/47 (46.81%)	13/48 (27.08%)	9/72 (12.5%)	8/58 (13.79%)
Tigecycline	16/52 (30.77%)	6/21 (28.57%)	1/59 (1.69%)	2/58 (3.45%)
