# Supplementary material for: Distribution and Antimicrobial Resistance Patterns of Critical and High-Priority Gram-Negative Pathogens Isolated from Endotracheal Aspirates in an Intensive Care Unit
Source: Microorganisms. 2026 Oct 7;14(10):2281. doi: 10.3390/microorganisms14102281 (PMC13707979; doi:10.3390/microorganisms14102281)
Supplement: Supplementary file 1 [file microorganisms-14-02281-s001.zip › Table S5.pdf]

Table S5. Antimicrobial resistance pattern of the *Klebsiella pneumoniae* isolated from endotracheal aspirates, 2022-2025

Antimicrobial agent	**2022** (n=50)	**2023** (n=112)	**2024** (n=58)	**2025** (n=51)
Ceftazidime	27/40 (67.5%)	69/110 (62.72%)	32/58 (55.17%)	14/42 (33.33%)
Ceftriaxone	21/43 (48.84%)	58/108 (53.70%)	28/56 (50%)	17/50 (34%)
Cefepime	19/47 (40.42%)	40/76 (52.63%)	21/49 (42.85%)	11/42 (26.19%)
Imipenem	13/44 (29.54%)	11/25 (44%)	18/48 (37.5%)	23/48 (47.92%)
Meropenem	15/47 (31.91%)	54/109 (49.54%)	24/58 (41.38%)	18/45 (40%)
Ciprofloxacin	20/45 (44.44%)	55/100 (55%)	30/57 (52.63%)	19/46 (41.30%)
Levofloxacin	17/44 (38.63%)	52/97 (53.60%)	27/50 (54%)	14/28 (50%)
Piperacillin/ tazobactam	18/46 (39.13%)	69/107 (64.48%)	27/45 (60%)	24/48 (50%)
Colistin	1/39 (2.56%)	5/105 (4.76%)	1/56 (1.78%)	0/30 (0%)
Gentamicin	20/43 (46.51%)	43/104 (41.34%)	19/57 (33.33%)	15/47 (31.91%)
Tigecycline	10/44 (22.72%)	8/27 (29.62%)	4/28 (14.28%)	4/29 (13.79%)
