# Supplementary material for: Distribution and Antimicrobial Resistance Patterns of Critical and High-Priority Gram-Negative Pathogens Isolated from Endotracheal Aspirates in an Intensive Care Unit
Source: Microorganisms. 2026 Oct 7;14(10):2281. doi: 10.3390/microorganisms14102281 (PMC13707979; doi:10.3390/microorganisms14102281)
Supplement: Supplementary file 1 [file microorganisms-14-02281-s001.zip › Table S6.pdf]

Table S6. Antimicrobial resistance pattern of the *Pseudomonas aeruginosa* isolated from endotracheal aspirates, 2022-2025

Antimicrobial agent	**2022** (n=99)	**2023** (n=103)	**2024** (n=97)	**2025** (n=123)
Ceftazidime	75/97 (77.32%)	47/99 (47.47%)	54/94 (57.44%)	65/111 (58.56%)
Cefepime	54/93 (58%)	34/72 (47.22%)	54/92 (58.69%)	63/110 (57.27%)
Imipenem	40/85 (47.05%)	56/98 (57.14%)	55/79 (69.62%)	84/119(70.58%)
Meropenem	55/98 (56.12%)	32/101 (31.68%)	51/93 (54.84%)	70/119 (58.22%)
Ciprofloxacin	57/96 (59.37%)	34/100 (34%)	50/92 (54.34%)	48/101 (47.52%)
Levofloxacin	33/65 (50.77%)	33/86 (38.37%)	53/93 (56.99%)	18/41 (43.90%)
Piperacillin/ tazobactam	45/96 (46.87%)	44/100 (44%)	47/94 (50%)	59/117 (50.42%)
Colistin	2/81 (2.47%)	7/98 (7.14%)	3/95 (3.15%)	3/66 (4.54%)
Gentamicin	48/87 (55.17%)	34/102 (33.34%)	47/95 (49.47%)	54/100 (54%)
Tobramycin	43/88 (48.86%)	20/94 (21.27%)	47/95 (49.47%)	60/117 (51.28%)
